# Work ability and percentage of hours worked related to limitations in patients with upper extremity musculoskeletal disorders: a cross-sectional cohort study

**DOI:** 10.1186/s12891-020-03387-y

**Published:** 2020-06-18

**Authors:** A. van Schaaijk, K. Nieuwenhuijsen, M. H. W. Frings-Dresen

**Affiliations:** Amsterdam UMC, University of Amsterdam, Coronel Institute of Occupational Health, Amsterdam Public Health research institute, Meibergdreef 9, PO Box 22660, 1100 DE Amsterdam, the Netherlands

**Keywords:** Functioning, Limitations, Repetitive strain injury (RSI), UEMSD, Work ability appraisal, Working hours, WRUED

## Abstract

**Background:**

The aim of this study was to assess the relationship between self-reported work ability and hours worked at the current time in Upper Extremity Musculoskeletal Disorders (UEMSD) patients. To further investigate this relationship, the association of work ability and working hours with several limitations in daily and working life were explored.

**Methods:**

In this cross-sectional cohort study, a questionnaire was sent out to members of the UEMSD patient organisation, containing self-reported work ability, questions on working hours and limitations in work due to UEMSD. Limitations were measured with the Disabilities of Arm Shoulder and Hand questionnaire, ShortForm-36 subscales, and common hand grasps or grips. Work ability was measured with the work ability score, while worked hours were operationalised as the percentage of hours worked compared to fulltime. The correlation between worked hours and work ability was tested with the Pearson correlation coefficient. Variance in work ability and the hours worked were explained by limitations and assessed with two linear regression analyses.

**Results:**

Based on data of 794 respondents a moderate correlation was found between work ability and worked hours r = 0.46; 95% CI [0.40, 0.53]. Models including limitations explained 52 and 21% of total variance in work ability and worked hours, respectively. Variance in both can be explained by the degree of difficulties performing daily activities at work, limitations in daily activities as a consequence of health issues and the ability to perform a precision grip. Additionally, work ability can be explained by limitations at work and other daily activities due to physical health issues, while the percentage of hours can additionally be explained by the ability to grasp a large object with one hand, the ability to use a keyboard, and the subject’s gender.

**Conclusions:**

The number of worked hours does not fully match the work ability. Although they share three predictors, work ability and worked hours seem to be based on different aspects. Compared to work hours, work ability is more strongly related to limitations in daily activities and work. Taking self-reported work ability into account can improve the fit between work limitations and work hours.

## Introduction

Work ability reflects the extent to which people can do their job satisfactorily with respect to their job demands and their (physical and mental) health [1]. Work ability is an important factor in working life and is used as an indicator for the ability to perform work at a specific time, and can predict the future need for a work disability pension [2–7]. Although work ability is an abstract concept, it is seen as a standard for determining whether someone is able to work and is often used as a marker for the current ability of that person to perform in a job [8–10]. The predictive value of work ability has been extensively studied, however, the relationship with the current situation is less well-known. The relationship between the self-reported work ability, and the actual ability to work several hours at that same timepoint remains unknown.

Work ability can be determined in several ways. One of the ways to assess work ability in practice is with job-specific testing in preventive medical examinations or observing work activities by occupational physicians [11]. Another more frequently used way to assess work ability is by letting workers appraise their own work ability [12]. In self-reported work ability, workers are asked to appraise their own work ability relating to their job demands at the current time. The first question of the Work Ability Index (WAI), also called the Work Ability Score (WAS), is often used for this purpose. The validity of the WAS has been established in relation to future sickness absences, but it is unclear whether the self-reported instrument WAS is a valuable and representative measure of what an employee can do at the current time in practice [5–7, 13]. Conceptually, one would expect a relationship between self-reported work ability and the actual hours that an employee has been able to do his/her job. If self-reported work ability is indeed representative of actual working hours, the WAS can aid occupational professionals in estimating the number of hours a worker can be required to work. When WAS and the hours worked do not concur, it is important to gain more information on why these measures differ and what causes the differences between the two estimates.

Workers with a chronic disease constitute a suitable population for studying the relationship between self-reported and actual work hours. Having a chronic disease with corresponding problems or limitations can lead to decreased mental and physical capacities, therefore threatening work ability and working hours [12, 14–21]. One such chronic condition is Upper Extremity Musculoskeletal Disorder (UEMSD) or Repetitive Strain Injury (RSI). In Europe, the one-year prevalence of neck and upper limb pain in the working population is almost 45% [22]. Although there is substantial variation in prevalence between countries, that number shows that at any given time, a major part of the workforce experiences musculoskeletal pain and accompanying limitations in the upper body. The pain and accompanying limitations performing daily tasks caused by (chronic) UEMSD during daily and working life can decrease the current work ability in UEMSD patients and cause absences due to illness [23].

To determine to what extent the self-reported work ability corresponds to the ability to actually work at the current time in a population of UEMSD patients, it is vital to assess to which extent self-reported work ability is related to the number of hours worked. Therefore, we aimed to assess the coherence between self-reported work ability and the hours worked at the current time.

To further study the potential differences between work ability and worked hours, experienced limitations during daily activities and work might be important. Limitations during daily activities requiring the use of the upper extremities can also indicate problems during work hours because most work is being performed with the use of arms and hands [24]. Gold et al. found that the highest prevalence of chronic UEMSD complaints are mild complaints. In the group of workers with mild complaints, 76% continued working [25]. Even in the most severe cases of chronic UEMSD, 50% of patients continued working. This means that a large group of chronic UEMSD patients is still engaged in work activities while experiencing complaints of the upper extremities, leading to limitations during daily activities. It is unknown to what extent these limitations influence both self-reported work ability and hours worked in a different way. Limitations occur when a worker is unable to perform a certain daily or work activity. These limitations determine the ability to meet certain job demands.

Insight into how limitations during work are related to work ability and worked hours is important because it can aid occupational professionals in adapting the work or workplace to better fit the ability to perform work of a worker with UEMSD complaints. However, it is still unknown what type of limitations during daily and work activities lead to decreased work ability and a decreased number of working hours in UEMSD patients. This indicates a need for studying work ability and the hours worked and their relation to UEMSD-related limitations. In summary, our aim is to assess the relationship between self-reported work ability and the hours worked at the current time and additionally assess how limitations in daily and work life can explain variance in work ability and the amount of hours worked in UEMSD patients. These aims lead to the following two research questions:

1. *What is the relationship between self-reported work ability and the hours worked at the current time in UEMSD patients? And*
2. *How are self-reported work ability and the hours worked at the current time related to limitations and activities in daily life?*

## Methods

### Design, participants, setting and procedure

In this cross-sectional study, a questionnaire was sent out in April 2005 to all 3250 members of the Dutch National UEMSD patient association, irrespective of the severity of complaints. Participants that were unemployed or on disability pension were excluded. Each questionnaire (‘RSI: Complaints, health and possibilities’) was accompanied by a participant information form. Filled out forms were returned anonymously and directly to the research institute by an enclosed return envelope. After 1 week, the members received a reminder to fill out and return the questionnaire. All patients participated on a voluntary basis and data was stored anonymously. The study was conducted in accordance with the declaration of Helsinki [26]. The research proposal for this secondary analyses of the data was submitted to and approved by the Medical Ethical Committee of the Academic Medical Center, which decreed that a comprehensive evaluation was not required as this study was not subject to the Medical Research Involving Human Subjects Act (W19_211#19.256).

### Instruments

Respondent characteristics and basic information were gathered by a questionnaire. Besides the basic information about work, several instruments were used to measure the work hours, complaints and the work ability of UEMSD-patients [27]. The questionnaire further contained questions on (health) complaints, health and health perceptions, work and limitations [28].

The primary outcome, self-reported work ability, was measured with the Work Ability Score (WAS), the first question of the Work Ability Index (WAI). This single item WAI was included to assess the appraised work ability at the current time (according to the patient) on a scale of 0 to 10. This single item question showed sufficient convergent validity to the complete WAI [29]. A score of 0 meant *not being able to work* and a score closer to 10 meant increasing work ability compared to *their lifetime best* (score 10). Hours worked, was operationalized as the percentage of hours worked compared to fulltime employment (36 h). To calculate this variable, participants filled out the number of hours they actually worked in the past week. This value was chosen rather than the percentage of worked hours compared to the contract hours because multiple respondents were on a 0-h or flexible contract basis while working multiple hours. Many other respondents worked a numerous amount more than their contract hours, leading to abnormal values, which made the contract hours unsuitable for these analyses.

To answer our second research question, the limitations were operationalised in three ways: The degree of difficulties in performing daily activities in the last week was assessed by the complete Disability of the Arm, Shoulder and Hand (DASH) questionnaire, including the work and sport module [30, 31]. The DASH scores are scaled with 0 indicating no disability and 100 indicating most disability.

A second way to assess limitations was to ask whether patients have problems performing certain frequent grips of both hand and wrist. Illustrations of these common grasps and movements were included in the questionnaire, accompanied by a question on the limitations of performing these in daily work life. These illustrations are shown in the Appendix. These grasps and activities were, among others, the cylindrical grip, the lateral key grip, the precision grip or spherical grip, the hook grip or medium wrap, and common activities at work (i.e. using a computer mouse or keyboard, pushing with the hand or finger, and bending the wrist) [27, 32]. An example question next to an illustration was: When you have to perform activities during work using your hand as displayed in the illustration above, do you experience problems? The answer options to these questions were: ‘Yes, almost always’, ‘Yes, sometimes’, ‘No, no problems’, or ‘No, I don’t have to do this’. The outcome was divided into either ‘yes, almost always’ scored as 1, and all the other answer options grouped together scored as 0. This means that a score of 1 indicates problems with performing this movement compared to ‘no’ or ‘only sometimes’ problems, which was coded as 0.

The third way was to assess limitations in the health-related quality of life by using the Short-Form-36 (SF-36) [33–35]. Of the nine subscales, only ‘physical functioning’, ‘pain’, and ‘physical role functioning’ were used in the analysis. The subscale of physical functioning measured ‘limitations in daily activities as a consequence of health issues’ during the last 4 weeks. The pain and physical role functioning subscales represent ‘pain and limitations due to pain’ and ‘limitations in work and other daily activities due to physical health issues’, respectively [35]. These scores are measured on a scale between 0 and 100, where a score closer to 100 indicates a better general health status. Averages for a Dutch population aged 35–44 were 90 (±14.4), 84 (±21.7) and 83 (±32.0) for physical functioning, pain and physical role functioning, respectively [35].

Additionally, demographic variables, among which age and gender were included, were used in the analyses to describe the study population and differentiate possible influencing characteristics. Males were coded as 1 where females were coded as 0.

### Statistical analyses

Before the analyses were performed, cases with over 50% missing variables were deleted. Cases with one or both primary variables missing were also deleted from the analyses. Before the analyses, the MCAR test was executed to establish the randomness of missing values. If the MCAR test was significant, no imputation has taken place. First, the self-reported work ability was related to the percentage of hours worked using a Pearson correlation analysis. In this analysis, the coherence between both outcomes was analysed.

Second, the variables, SF-36 subscales, DASH scores, the limitations in frequent grasps of hand and wrist, and age and gender were related to both self-reported work ability, and worked hours. In these analyses, the self-reported work ability and percentage of hours worked were entered as dependent variables, and the SF-36 subscales, the DASH scores and the limitations in frequent grasps of hand and wrist were included as independent variables. Two separate models were created, one for self-reported work ability and the other for the percentage of worked hours compared to fulltime employment, with only significant contributors to the explained variance. This makes assessing the limitations that explain the most variance in work ability and the percentage of hours worked possible. In this way, the most probable limitations that cause people to not be able to fully function in their job can be identified. Variance in work ability and the hours worked were explained by these limitations and assessed with two linear regression analyses. A model was created using backward selection. Non-significant variables were sequentially deleted from the model, creating a new model, until all remaining variables in the model had a *p*-value smaller than 0.05, and the model was complete.

As a final check, we tested both models for heteroscedasticity (through a visual inspection of the residual plots) and collinearity (through collinearity statistics: tolerance and Variance Inflation Factor (VIF) score). A tolerance ≤0.4 and a corresponding VIF score over 2.5 was set as cause for concern of collinearity, with tolerance calculated as 1-R^2^ and VIF as 1/tolerance. The statistical analyses were done performing multiple regression analyses in IBM SPSS Statistics version 25.

## Results

### Population characteristics

**Fig. 1:**
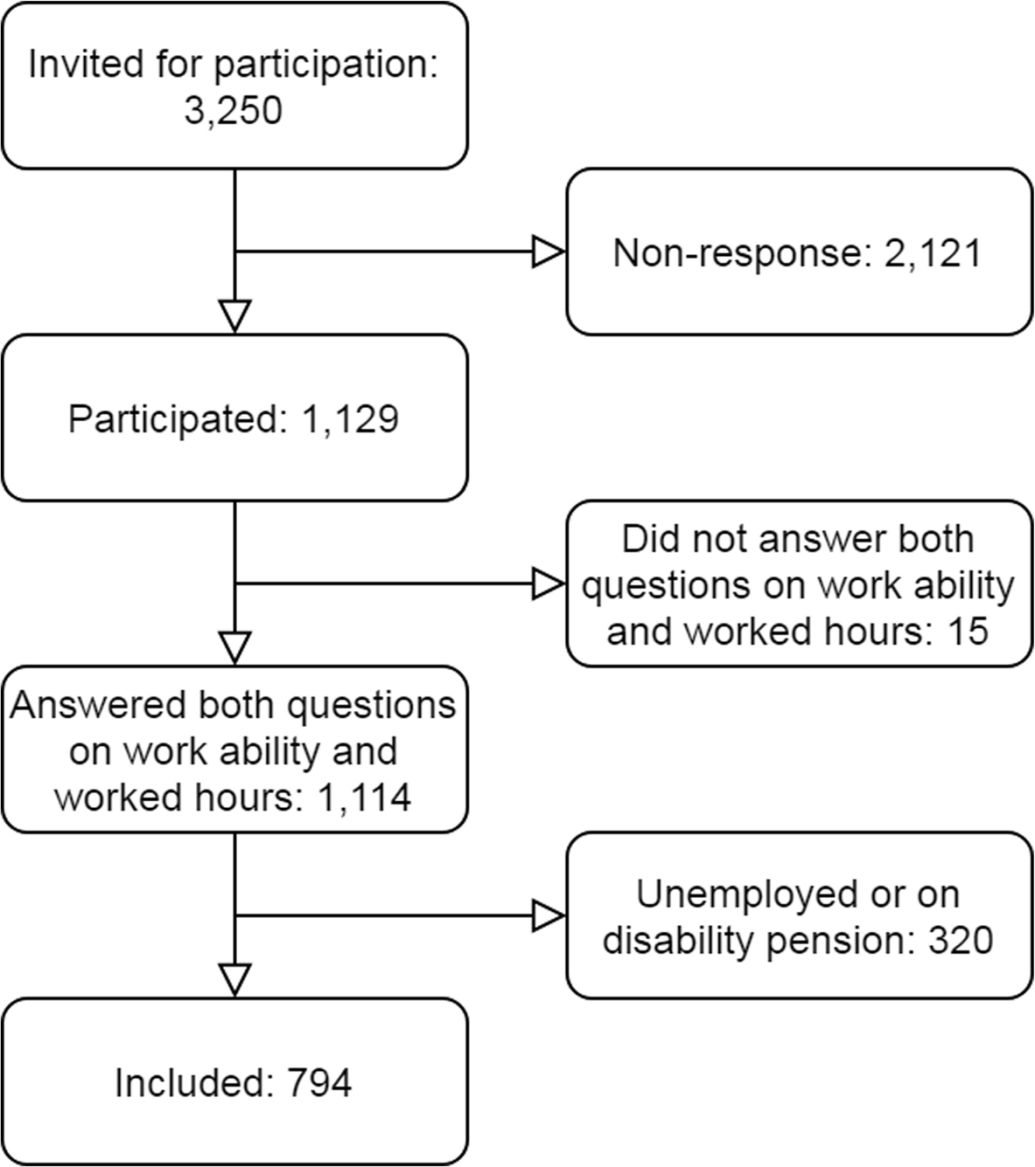
Flowchart of inclusion process and number of included participants

In total, 1129 UEMSD patients responded (35%), of which 794 were eligible for secondary analyses as they filled out both questions regarding work ability and hours worked and were currently employed [28]. Individuals who received disability pension, or did not have a job were excluded from the sample. The inclusion flowchart is shown in Fig. 1. The MCAR test showed that some missing values are not randomly missing. As a result, no imputation took place, and no data is listwise deleted.

The mean age at the time of participation was 40.0 (±8.5) years and one-third of all participants were male. Over 90% of included participants reported they were still working. The mean duration of complaints was 5.6 (±3.1) years, and the mean extensiveness of pain regions was 5.8 (±3.6) on a scale of 0 to 16 regions. Participant characteristics (of all respondents) are shown in Table 1. Population characteristics divided per job category according to the International Standard Classification of Occupations [36] are shown in the Appendix.

**Table 1:** Percentage or mean ± SD of participant characteristics of work ability and percentage of hours worked (*n* = 794)

	%	**Mean**	**SD**	***N***
Age at time of participation		40.0	8.5	794
Gender male	37%			794
Education level				794
Primary school	1%			
Lower vocational education	1%			
Secondary vocational education	26%			
Higher vocational education	34%			
University	32%			
Other	6%			
% participants with pain	80%			794
% participants with stiffness	49%			794
% participants with tingling feelings or numbness	42%			794
Main cause of the start of complaints				742
A single cause cannot be identified	17%			
Overload during leisure	2%			
Short-term overload during work	5%			
Long-term overload during work	70%			
Accident, strain	1%			
Other(e.g. stress, posture, furniture)	53%			
Complaint duration in years		5.6	3.1	755
Work ability score (WAS) (0–10)		6.4	2.0	794
% worked of Fulltime employment (0–100)		77.0	35.0	794
DASH score (0–100)		28.3	19.0	668
DASH work module (0–100)		41.0	25.8	784
SF-36 subscale physical functioning (0–100)		80.0	15.3	780
SF-36 subscale pain (0–100)		59.3	20.1	793
SF-36 subscale physical role functioning (0–100)		41.9	39.3	788

The percentage of hours worked correlates significantly (*p* < 0.001) with the self-reported work ability (*n* = 794). The correlation coefficient is *r* = 0.46; 95% CI [0.40, 0.53] *p* < 0.001, which is considered a modest or moderate correlation [37].

Results of the univariate analyses are shown in the Appendix. In the multivariate analyses, the prediction model for the WAS contained four variables. These variables were able to explain 52% of total variance in WAS. The DASH work module score, the SF-36 physical functioning and physical role functioning domains and the precision grip were predictors in this model. Multicollinearity was not of concern in the WAS model as there were no VIF-scores over 2.5.

The model that explained 21% of total variance in percentage of hours worked contained five variables, namely: SF-36 physical functioning domain, the DASH score, the DASH work module, limitations using a keyboard and gender. Multicollinearity was a concern in the worked hours model where the VIF-score of the DASH was 3.4. When this variable was removed from the model, there was no more concern for collinearity. However, the variables ‘ability to hold a precision grip’ and ‘ability to grasp a large object with one hand’ became significant contributors and were added to the model again in accordance with the methods. The explained variance of the new model, without the DASH score, but including the re-entered variables as predictors, is also 21%. A higher score of the B-coefficient means a more positive contribution to the work ability score. Both final models are displayed in Tables 2 and 3. A plot of the residuals was analysed for a check on heteroscedasticity, but there were no reasons to believe this was of concern in either model.

**Table 2:** Model with statistically significant predictors of explained variance in WAS

Model		Unstandardized Coefficients		*p*-value	95% Confidence Interval for B	
		B	Std. Error		Lower Bound	Upper Bound
1	(Constant)	5.487	0.592	< 0.001	4.322	6.652
	DASH work module	−0.034	0.004	< 0.001	−0.042	− 0.025
	SF36 physical functioning	0.025	0.006	< 0.001	0.014	0.037
	SF36 physical role functioning	0.008	0.003	0.002	0.003	0.014
	Precision grasp	−0.489	0.215	0.024	−0.913	− 0.065

Explained variance of this model: R^2^ = 0.519

**Table 3:** Model with statistically significant predictors of variance in worked hours

Model		Unstandardized Coefficients		*p*-value	95% Confidence Interval for B	
		B	Std. Error		Lower Bound	Upper Bound
1	(Constant)	52.439	11.561	< 0.001	29.725	75.153
	DASH work module	−0.418	0.068	< 0.001	− 0.550	− 0.285
	SF36 physical functioning	0.318	0.115	0.006	0.092	0.544
	Precision grip	13.007	4.210	0.002	4.737	21.278
	Grasping a large object with one hand	−9.092	4.622	0.050	−18.174	−0.011
	Using a keyboard	13.539	5.020	0.007	3.677	23.402
	Gender	14.366	3.030	< 0.001	8.412	20.320

Explained variance of this model: R^2^ = 0.207

In this model, a higher SF-36 score, having problems with using a keyboard, having problems performing a precision grip and being male resulted in a higher percentage of worked hours, while a higher score on the DASH work module and limitations in grasping a large object with one hand predict a lower percentage of worked hours in individuals. In the univariate analyses, limitations using a keyboard and the ability to hold a precision grip are not significant. However, in the multiple regression, these variables become significant indicating suppression effects (or negative confounding) between variables. Limitation in using a keyboard is unsuppressed by the presence of the DASH work module.

## Discussion

Self-reported work ability and the percentage of hours worked correlate moderately, with a higher work ability score corresponding to a higher percentage of hours worked. Despite this moderate correlation, work ability and worked hours are explained to a different extent by limitations. Explained variance by limitations is higher for work ability than for worked hours. The degree of difficulties performing daily activities in work (DASH work module), the limitations in daily activities as a consequence of health issues (SF-36 physical functioning) and limitation in performing a precision grasp or grip explain the variance for both self-reported work ability and worked hours. The limitations in work and other daily activities due to physical health issues (SF-36 physical role functioning) explain variance in the self-reported work ability model, but not in the model of percentage of worked hours. Variance in the percentage of worked hours is additionally explained by the ability to grasp a large object with one hand, limitations in using a keyboard and gender.

The WAS may be informative when establishing a reintegration plan since its relation to limitations is stronger than for the number of worked hours at the current time. Variance in the current work hours are only modestly explained by variables. The work hours do not seem related to many aspects of work and health included in our study. However, the WAS is an individual’s estimation of their ability to meet their job demands that takes the limitations that an employee experiences into account. Consulting WAS can help to better match work hours to the limitations in work and daily life in order to prevent sick leave. By monitoring WAS, it is possible to timely adjust working hours as a preventive measure.

Gender did not explain variance in work ability in this population, while it did play a role in the estimation of working hours. Male participants have a 14% point higher percentage of working hours when experiencing the same limitations. The difference between men and women in the percentage of working hours might be explained by the fact that the overall number of women working fulltime is lower—28% vs. 58% of men. In this study, fulltime was set at 36 h a week. The difference between men and women might be explained by the fact that more women work part-time than men [38]. This, in turn, can explain the difference in explained variance by gender in the number of hours worked at the current time and the lower mean percentage of worked hours in women.

### Comparison with other studies

Work ability has not been related to current work hours in populations with limitations in studies before. We could not identify other studies that studied this specific relationship. Leijten et al. (2014) did find that WAS and productivity at work (0–10), a concept closely related to working hours, were positively correlated in older employees with chronic diseases (r = 0.23, *p* < 0.01) [39]. Whereas Leijten studied the influence of chronic limitations on work ability 1 year later, our study provides knowledge on limitations at the current time in relation to work ability and worked hours at the same moment. Another study by Oakman et al. (2019) studied the relationship between the number of pain sites and work ability trajectories [40]. They found that the number of pain sites was associated with a decreasing work ability trajectory over time. A study by Kamaleri et al. (2009) showed the strong predictive power of the number of pain sites on work disability 14 years later [41]. In our study, pain was included as a potential predictor of workability, but was not retained in the final model. This means that not only pain predicts work ability, but that limitations in work might help better predict current work ability. However, these limitations are only moderately related to the hours worked. Although future work disability and current work ability represent different concepts, we would argue—based on our study—that not only the number of pain sites or number of limitations is important, but also the type of limitations in cohesion with the type of work.

### Strengths and limitations

A strength of this study is that we studied a population with a large variation in work ability due to the existence of limitations/health problems during work and daily life. This study population showed a larger variance in WAS in comparison to a general working population [42]. When studying the coherence between work ability and hours worked, a population with a large variation in work ability would be beneficial in order to study correlations between the two variables.

A limitation of this study is that the type of work that participants do was not taken into account. However, given the fact that participants have UEMSD complaints, it is plausible that the work of these participants involves working with arms and hands. All questions regarding movements and hours concerned the current job of the respondent, as is the assessment of self-reported work ability. If participants did not have to do certain activities during work, these were regarded as not being a limitation, and therefore not affecting work, and this should therefore not be a large source of bias. However, we expect some selection bias. Although the entire population of the UEMSD patient association received an invitation to participate in this study, it can be expected that the more severe cases were represented more strongly in the UEMSD patients association.

An aspect that might explain the difference in explained variance by limitations between work ability and the percentage of hours worked is the common method principle due to the cross-sectional nature of this study [43]. This principle suggests that two measurements are more strongly related to each other than a third variable due to the type of measurement. In this case, the limitations and WAS are both assessed by a questionnaire filled in by the workers themselves. The number of hours, however, is more factual and not only determined by the workers themselves, as the occupational physician and their employer play a role in determining the optimal number of work hours.

The outcome of hours worked was used to represent the impact of UEMSD-related limitations on the ability to perform work. However, since the variance in worked hours is only explained to a moderate extent by the factors in our model, we can conclude that hours worked is influenced by other factors than just these limitations. The family situation, social security system, psychological state, need for income generation or informal care can all influence the reason to work [44]. This can explain the moderate correlation between work ability and the hours worked at the current time.

### Implications

It seems that the number of hours worked does not fully match with self-reported work ability. When determining the work hours of a UEMSD patient, it can be profitable to better align these work hours with work ability, as workers take their limitations at work into account when assessing their own work ability. In this way, the working hours of a UEMSD patient correspond better with their self-reported ability to meet the job demands. Limitations are individual and job-specific. Workers can estimate the influence of limitations on their work best because they know every details about their job demands in relation to the limitations they encounter. Therefore, the appraisal of their work ability can be used to better match work hours to their limitations in order to prevent workers from going on sick leave. In the Netherlands for instance, an occupational physician can be consulted when a worker is unable to meet the work demands. This occupational physician advises in consultation with the employee and employer how and if work can be continued. One potential outcome of the consultation is that the employer is advised to change the work demands or work hours. When this proves impossible, the occupational physician can advise other jobs within or outside the company, that can be met by the employee without compromising their health.

Occupational physicians can use the appraisal of UEMSD patients’ work ability to better match the ability to meet job demands with the working hours, keeping the limitations in mind. Occupational physicians can advise their patients to have their work hours and demands match their abilities. The results of this study are relevant for occupational professionals such as occupational physicians as they give an indication of which functional limitations affect the work ability most, for instance the ability to hold a cylindrical grip, precision grip and hook grip. The importance of the capability to perform certain actions can aid occupational professionals in determining the next steps in the treatment, to decrease the impact of limitations on work life. To confirm the additional value of taking work ability into account when determining work hours, a longitudinal study could be performed. Based on the univariate analyses, limitations in bending the wrist are quite important in some individuals, but not significant on a group level. For which individuals this limitation is of importance needs to be studied in future research. Future research can focus on differences between types of job demands. A division can be made between more physical demands using shoulders, or more desk work using fingers and hands. With this information, future work disability or sick leave might be prevented by using the right strategies to ensure work hours match work ability. Furthermore, the ability to use a keyboard, the ability to hold a precision grip and the ability to grasp a large object with one hand are only related to the amount of hours worked when the DASH work module was present in the final model, and the B-coefficient changed direction. Since this indicates paradoxical confounding, the relationship between these variables needs to be investigated further.

## Conclusions

Self-reported work ability is moderately correlated with the percentage of hours worked at the current time. Variance of both aspects is explained by limitations in a different way. Work ability is more strongly related to limitations in work than work hours themselves. Taking self-reported work ability into account can improve the fit between work limitations and work hours.

## Figures and Tables

**Fig. 2:**
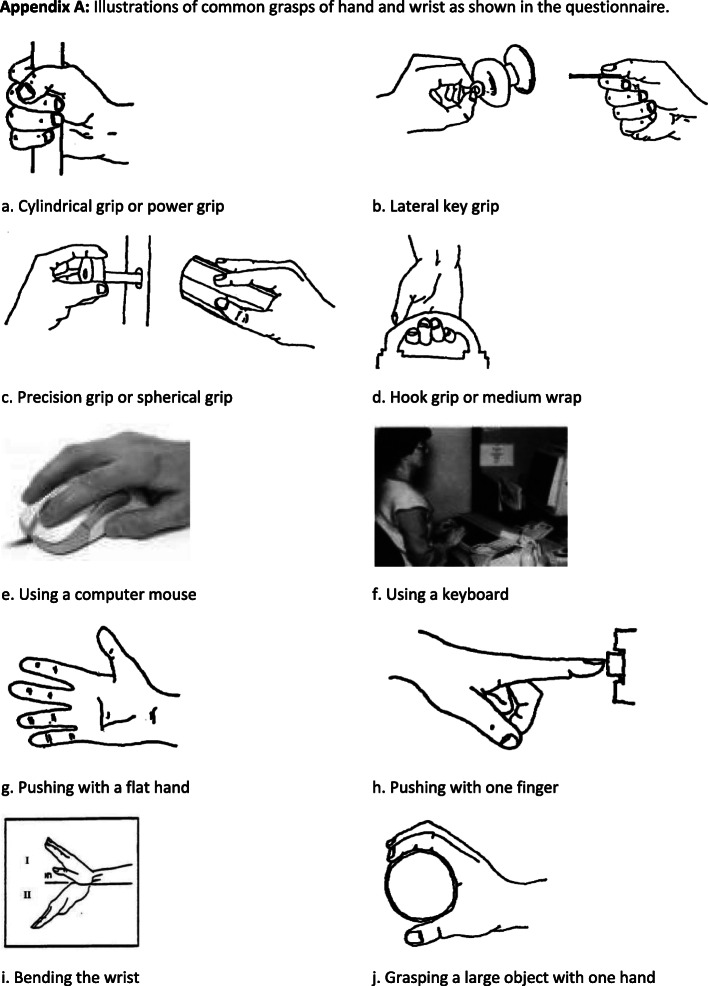
Illustrations of common grasps of hand and wrist in the questionnaire; When you have to perform activities during work using your hand as displayed in the illustration above, do you experience problems? The answer options to these questions were: ‘Yes, almost always’, ‘Yes, sometimes’, ‘No, no problems’, or ‘No, I don’t have to do this’. (Illustrations derived from the questionnaire ‘RSI: Complaints, health and possibilities’ with permission to publish)

## Data Availability

Data are available at the first author upon reasonable request.

## Appendix

**Table 4:** Results of the univariate analyses of explained variance in Work Ability and hours worked

*Variable*	Work Ability Score (0–10)			Percentage worked of fulltime (0–100)		
	*p-value*	*Adj R*^*2*^	*B*	*p-value*	*Adj R*^*2*^	*B*
Cylindrical grip	< 0.001	0.12	−1.69	< 0.001	0.04	−16.53
Lateral key grip	< 0.001	0.09	−1.58	0.003	0.01	−11.41
Precision grip	< 0.001	0.10	−1.61	0.062	0.00	−6.95
Hook grip	< 0.001	0.10	−1.36	< 0.001	0.03	−12.45
Using a computer mouse	0.001	0.02	−1.04	0.437	0.00	4.362
Using a keyboard	0.002	0.02	− 0.92	0.683	0.00	2.16
Pushing with a flat hand	< 0.001	0.07	−1.51	0.008	0.01	−10.84
Pushing with one finger	< 0.001	0.02	−0.91	< 0.001	0.02	−7.68
Bending the wrists	0.082	0.14	−2.44	0.279	0.02	−20.15
Grasping a large object with one hand	< 0.001	0.07	−1.43	< 0.001	0.03	−17.88
DASH	< 0.001	0.36	−0.06	< 0.001	0.09	− 0.56
DASH work module	< 0.001	0.42	−0.05	< 0.001	0.08	−0.39
DASH hobby/sport module	< 0.001	0.20	−0.03	< 0.001	0.05	−0.26
SF-36% Physical functioning	< 0.001	0.25	0.07	< 0.001	0.10	0.75
SF-36% Physical role functioning	< 0.001	0.29	0.03	< 0.001	0.06	0.22
SF-36% Pain	< 0.001	0.34	0.06	< 0.001	0.07	0.48
Age in 2005	0.008	0.01	−0.02	0.035	0.00	−0.31
Gender	0.002	0.01	0.45	< 0.001	0.08	20.02

**Table 5:** ISCO classification of participants for males and females

	Female		Male		Total	
	*N*	Percent	*N*	Percent	*N*	Percent
Cat 1. Managers	59	11.8	44	15.0	103	13.0
Cat 2. Professional	78	15.6	51	17.4	129	16.2
Cat 3. Technicians and associate professionals	18	3.6	67	22.9	85	10.7
Cat 4. Clerical support workers	205	40.9	76	25.9	281	35.4
Cat 5. Service and sales workers	44	8.8	19	6.5	63	7.9
Cat 7 or 8. Craft and related trades workers & plant and machine operators, and assemblers	64	12.8	20	6.8	84	10.6
Student	8	1.6	8	2.7	16	2.0
Not classifiable	25	5.0	8	2.7	33	4.2
Total	501	100	293	100	794	100

## Abbreviations

WAI: Work Ability Index
WAS: Work ability score
RSI: Repetitive Strain Injury
UEMSD: Upper Extremity Musculoskeletal Disorders
DASH: Disability of the Arm Shoulder and Hand
SF-36: Short Form 36
